# Death by Chloroform

**Published:** 1899-10

**Authors:** 


Death by Chloroform.—At Leeds, on June 23d, an inquest was held with respect to the death of a page boy, aged seventeen years, who had been employed by Dr. F. B. Musgrave, first as a surgery boy, and afterward as assistant dispenser. He was found dead in bed, with a bottle, which had contained chloroform, by his side, and a piece of cotton-wool covering his face. Evidence was given to the effect that the boy had suffered from neuralgia and toothache, and it was supposed that he had used the chloroform to alleviate pain. Death was attributed to mechanical suffocation, and the jury returned a verdict of death by misadventure.—*Pharmaceutical Journal.*

